# The street as transdisciplinary infrastructure: real world labs in One Urban Health—*well-being corner shops in Québec, Canada*

**DOI:** 10.1186/s44263-025-00235-w

**Published:** 2026-01-07

**Authors:** Evelyne de Leeuw, Cécile Aenishaenslin, Malek Batal, Olivier Beauchet, Michèle Bouchard, Antoine Boudreau LeBlanc, Katherine Frohlich, Yan Kestens, Sébastien Lord, Louise Potvin, Yves Terrat, Juan Torres, Martin Trépanier, Noémie Harriet

**Affiliations:** 1 Department of Social and Preventive Medicine, School of Public Health, Université de Montréal, 7101 Avenue du Parc, Montreal, QC H3N 1X4 Canada; 2 One Urban Health, Sydney, Australia; 3 Urban Health & Policy, UNSW Sydney, Sydney, Australia; 4 International Union for Health Promotion and Education (IUHPE), Montréal, Canada; 5 Department of Pathology and Microbiology, Faculty of Veterinary Medicine, Université de Montréal, Montréal, Canada; 6 Department of Nutrition and Department of Social and Preventive Medicine, Université de Montréal, Montréal, Canada; 7 Department of Medicine, Université de Montréal/Institut Universitaire de Gériatrie de Montréal, Montréal, Canada; 8 Neurology/Geriatrics, Université de Montréal/Institut Universitaire de Gériatrie de Montréal, Montréal, Canada; 9 Department of Environmental and Occupational Health, Université de Montréal, Montréal, Canada; 10 Université de Montréal, Montréal, Canada; 11 CIHR Institute of Population and Public Health, Université de Montréal, Montréal, Canada; 12 CIHR Urban Interventions and Population Health, Université de Montréal, Montréal, Canada; 13 School of Urban Planning and Landscape Architecture, Université de Montréal, Montréal, Canada; 14 Ivanhoé Cambridge Observatory, Université de Montréal, Montréal, Canada; 15 CReSP, Université de Montréal, Montréal, Canada; 16 Nature & Life, Montréal, Canada; 17 Digital Health Consortium, Université de Montréal, Montréal, Canada; 18 Department of Mathematics and Industrial Engineering, Polytechnique Montréal, Montréal, Canada; 19 CIRRELT, Polytechnique Montréal, Montréal, Canada

**Keywords:** Urban, Wellbeing, Street, Nucleus, Transdisciplinary, Québec

## Abstract

Urban environments are places where challenges and opportunities associated with One Health happen. After briefly describing the evolution of cities and urban planning, and the One Health context, the piece seeks a heuristic to focus on particular issues at their interface: One Urban Health. The concept of One Urban Health is defined as an integrated, unifying approach that recognises the pivotal role of urban cultural, social and physical environments to sustainably balance and optimize the health of ecosystems, animal and plant communities, and individual humans, their groups and communities equitably. It recognizes that health and wellbeing of humans, domestic and wild animals, plants, and the wider environment (including ecosystems) are closely linked and networked and inter-dependent intergenerationally, socially and institutionally. Out of a potential myriad issues, populations, places and phenomena this paper identifies a place-based governance perspective for One Urban Health. The governance gaze applied here is multi-level governance, a pre-condition for effective (one urban health) policy making. The particular urban place where governance across and for human and ecological health communities is established is the street: both a location, a connection, and a meaning. This aligns with a number of traditions in urban health planning, including street-level bureaucracy and street science. A natural sequel, then, is the identification of nucleation entities and phenomena (things that are the nucleus of change) that are characterised as ‘Real World Laboratories’. In the Québecois context, these would achieve the shape of Well-Being Corner Shops (‘Dépanneurs de Recherche’) or urban socio-ecological development nuclei.

## Background

### One Health

Cities have become the dominant piece of anthropogenic infrastructure. Some would say that the emergence of cities as a form of human settlement from at least 15,000 years ago has been ‘organic’, that is, following natural patterns of growth [1]. In this article, however, we identify a dearth of focus on natural dimensions in urban systems. This lack of attention extends to conceptualisations of One Health (traditionally, an approach driven by concerns about zoonoses, antimicrobial resistance and food safety; more modern conceptualisations include broader patterns of pathogenesis including toxins and plastic as well as climate change; and the newest iteration sees One Health in cities as a key determinant of health promotion. The prevalent current definition as codified by the One Health High Level Expert Panel OHHLEP is “… an integrated approach that aims to sustainably balance and optimize the health of people, animals, and ecosystems. It acknowledges the close links and interdependence between the health of humans, animals, plants, and the environment.” [2]).

### One Urban Health

Our proposal is to step away from a human-centred discourse to an ecological and planetary view. Such an integration of One Health into Healthy Cities could be badged ‘One Urban Health’. To understand its potential, we show that a pertinent research lens would embrace governance conceptualisations and practices. In order to move from abstract argument to operational action, we argue that a physical presence in the very quintessence of cities – its streets – is necessary to initiate, implement and sustain change. We suggest that mobile (community and ecological) health research and development (R&D) hubs are appropriate infrastructures to roam the arteries of human settlement—streets. The associated research gaze must necessarily be transdisciplinary [3] *(‘… a global approach to complexity (…) between, through and beyond traditional disciplines. (…) It helps us generate novel transformative propositions’* as per Renn [4]*).*

In a world of polycrises and increasingly complex relations between the global and local, and collapsing biodiversity and ecosystem services [3], transdisciplinarity is, in our view, essential [5]. Alvargonzalez [6] adopts Klein’s view [7] that ‘transdisciplinarity is transcending, transgressing, and transforming, it is theoretical, critical, integrative and restructuring but, as a consequence, it is also broader and exogenous’. In particular in the Francophone world, there is a strong and continually evolving tradition on transdisciplinarity at the interface of ecosystems, sustainability, and the living environment. Dominique Charron [8, 9] builds a theoretical and applied, transversal as well as glocal [10] perspective that allows for strong transformative agendas across the Academy and community. The transformation is not just societal and ecological – it changes, in fact, the nature of the discourse and the very boundaries across which transdisciplinarity moves [11, 12]. Clearly, such perspectives build on French speaking traditions around Actor-Network Theory (ANT) and the recognition of meaningful and complex intertwinedness between individual and institutional actors, issues and events (‘actants’), and the physical environments, i.e., landscapes and places, in which transformative change takes place and can be understood. Latour [13], Callon [14] and Pinson [15] continue to add depth to the understanding of transdisciplinary science in action. With the evolution of the modern One Health paradigm [2], the integrative, ecosystems-conscious and (ecological) community-driven perspective builds on the view that beyond-human elements (other species, places, contexts and landscapes) play profound roles [16]. Recognising the role of land, landscapes and places in a significant – often more broadly accepted Indigenous – cosmology (worldview) adds a more profound ecosystems transdisciplinary dimension [17, 18] to the discourse. This has consequences for our understanding of multi-level governance as a key transformative gaze which we will introduce below.

‘The city’ continues to be a growing flashpoint of global attention and contention. Cities are the dominant form of settlement demographically (we are an urban planet since about the year 2007 [19]). They also create significant planetary concerns (cities are vast engines of carbon emissions and consumption with large spatial and ecological footprints). Perhaps more importantly, there are social and sustainability threats and opportunities for all human and more-than-human (ecological and technological [20]) communities in and around urban systems. The COVID-19 pandemic showed how many local governments were adaptive and creative in attempting to enable and facilitate effective non-pharmaceutical interventions (NPIs) [21]. On the other hand, most were incapable of appreciating and addressing the mechanisms and pathways that enabled the virus to emerge, spread, and affect health and wellbeing. This is no great surprise: although the birth of ‘modern public health’ co-evolved with nineteenth century industrialisation and urbanisation patterns in the centres of colonial powers, actual public health monitoring and pandemic preparedness are not traditionally the prerogative of local government. These responsibilities are either attributed to distinctive agents not embedded in urban infrastructures (e.g., ‘chief medical officers’) or are found at other – higher—levels of government [22] or civil society.

Cities are complex ecosystems where biological systems and anthropogenic pressures – and predominantly due to anthropocentric decisions – change the landscape (hardware, such as roads, sewage systems and housing as well as software including institutions and behaviours at the cognitive and collective scale) intersect. Since the birth of cities in the Levant and the Indus Valley some 15,000 years ago, humanity has attempted to regulate and plan away perceived risks and uncertainties imposed by nature. This regulatory stance – requiring a degree of deliberate governance over lands, resources, and gatherings (see below) – started with ensuring food security and food supply, followed by sewage systems and vector control, and eventually spatial urban planning. The conceptualisation of the role, function and place of the city in a wider socio-ecological understanding also evolved. As Schouwenberg et al. [23] wrote: “*Confronted with resource scarcity, industrialisation and colonial exploitation, Europeans in the nineteenth century conceptualised and experienced the natural world in new ways. In scientific and political discourse, the notion emerged that man depended on a fragile and interconnected system, which needed to be used wisely and preserved in order to endure. Nineteenth-century ideas about ecological limits to growth, sustainable use of resources, and intrinsic links between the economy, society, and the natural environment provided the conceptual building blocks for the sustainability discourse which shaped environmental thinking in the twentieth century*.”

Uncontrolled industrial growth in most cases was at the detriment of sustainability and ecological balance. Some enlightened souls have always regarded the natural and built environment as inextricably connected. Hausmann in the mid-1800s, for instance, described his newly designed Paris sewer network as the intestines of the living city. Olmsted designed Central Park (New York) and Mont Royal (Montreal) as the lungs of the city. But these attempts see sanitised and designed ‘nature’ as a separate endeavour from other urban development, e.g., in social, health or commercial spheres. In this view, the city regulates, rather than integrates. Even ‘modern’ insights such as ‘Nature-Based Solutions for Cities’ [24] are *designed* rather than *organic*. In other words: are designed for human-centered control and stewardship instead of resilience and adaptation in a context of anthropological processes and ecological dynamics [1].

This is a conceptual as well as practical challenge. Not only may retaining an anthropocentric view of the urban planning process stand in the way of more inclusive and emancipated views of a planetary perspective (in which social, ecological and spiritual dimensions play important roles [25]), but there are profound arguments to be made to safeguard and increase, rather than control and limit, biodiversity in cities. Ecologists and epidemiologists have shown that environments with high biodiversity parameters increase ecosystem resilience (such as many of the “hotspot” Earth’s tropical zones), and thus the habitat stability for all species, but this biodiversity comes with a higher inter-species competition, which often expresses as the source of emerging zoonotic pathogens [26]. At the same time, the complex diversity of the system can also reduce the effect of those pathogens. On the other hand, less biodiverse systems such as cities, reduce the ecological habitat resilience and that capability to instantly control pathogenicity [27]. Of note, of course, is that pathogenicity affects all species and not just humans – in fact, more species are affected by humankind than the other way around, and many humans are wholly unaware of raging pandemics in the animal and plants worlds (e.g., the bacterium of *Xylella Fastidiosa* ravaging olive trees around the world – with severe economic and food system consequences) [28].

## A prism to address planetary urban complexities

The above scoping formulates a recognition that goes beyond the idea that Earth’s systems can be completely known and quantified. Instead, they are multiple interconnected and interdependent parts that interact in ways that produce emergent behavior—outcomes that cannot be easily predicted by apprising the individual components alone. Such systems are characterized by non-linearity, self-organization, adaptation, and feedback loops [1], mechanisms currently recognised as integral to complexity science. Recent years have seen increasing attention to such complex dynamics [29].

Driven by food safety concerns and attempts at control of antimicrobial resistance in the intensive bio-industry, and an increasing awareness that the encroachment of human populations and domestic animals on natural ecosystems favors the emergence of potentially zoonotic pathogens (including the cohabitation and consumption of a multitude of species, including chickens, pigs, bats and pangolins), the concept of One Health entered global political agendas. The *Quadripartite* (an alliance of United Nations Environment Programme (UNEP), World Health Organization (WHO), Food and Agriculture Organization of the United Nations (FAO) and World Organisation for Animal Health (WOAH)), with an associated One Health High-Level Expert Panel (OHHLEP) defined “*an integrated, unifying approach that aims to sustainably balance and optimize the health of people, animals and ecosystems. It recognizes the health of humans, domestic and wild animals, plants, and the wider environment (including ecosystems) are closely linked and inter-dependent*” as One Health [2].The purpose of expert panels in these high-level (United Nations (UN); global) realms is to set normative standards, but we acknowledge that much of the above definition is therefore potentially detached from reality on the ground (and in the street) [30]. It also may be interpreted as a normative statement from a ‘normal science’ gaze. The authors of this piece, in their long-term development program, interpret ‘integrated unifying’ as ‘place-based ecosystemic and dynamic’.

The ponderings and pronouncements of the Quadripartite and its OHHLEP often fail to identify *where* this integrated and unifying approach is to happen. Fortunately, the field of health promotion comes to our aid here. This field asserts that health (human; ecological; animal; natural; and cosmological) is created in settings [31], rather than in risk groups (which are traditionally associated with deficit based and disease epidemiology). The Ottawa Charter for Health Promotion (1986) [32] states that “*Health is created and lived by people within the settings of their everyday life; where they learn, work, play and love. Health is created by caring for oneself and others, by being able to take decisions and have control over one's life circumstances, and by ensuring that the society one lives in creates conditions that allow the attainment of health by all its members. Caring, holism and ecology are essential issues in developing strategies for health promotion*.” Following this early conceptualisation of places and spaces for health and wellbeing, a rich tradition of place-based interventions [33] grew. Cities, neighborhoods, parks, boroughs and precincts [34], and schools are such places, as are wetlands, pig sties, food laboratories and wet markets. Virtual environments and gathering places (like TikTok, BlueSky and LinkedIn) may have to be added to those environments where One Health plays out [20].

Of all these settings, cities have received probably the most prominent and enduring social, political and scholarly attention. Usually this has not been a level of focus that has included a broad (even holistic) emancipatory planetary health or One Health emphasis. The Canada Excellence Research Chair *One Urban Health* (CERC OUH) has been charged with providing a research and action agenda for the interface between ecosystemic and human health in cities. Specifically, we seek to understand implicit and explicit rules, regulations and conventions across levels of species and institutions that create and maintain that interface – this is ‘governance’ [35, 36].

As such, we define One Urban Health as.

*an integrated, unifying approach that recognises the pivotal role of urban cultural, social and physical environments to sustainably balance and optimize the health of ecosystems, animal and plant communities, and individual humans, their groups and communities equitably. It recognizes that health and wellbeing of humans, domestic and wild animals, plants, and the wider environment (including ecosystems) are closely linked and networked and inter-dependent intergenerationally, socially and institutionally.*

## Where the rubber hits the road: governance

‘One urban health’ problems have sometimes been framed as phenomena without distinctive localisation. Issues such as climate change, ecological or transportation injustice, or the urban macrobiome (the interconnecting communities and webs of all states of life, and their determinants, in urban environments), though often conceptualised in abstract terms, deserve concrete and place-based activist and scholarly engagement. The study of human and ecosystem health in cities must necessarily be located in particular places [37]. The scholarly and practical literature is rife with an enormous diversity of such places – they can be residential and office buildings (redesigned for urban farms [38]; or as intrinsic resources for vertical biodiversity [39]) or transportation infrastructure (a disused rail line [40], or demolished highway and urban regeneration [41]). They can be blue (rivers and their estuaries, lakes [42], fountains) and green space (as ecological corridors [40], food forests [43], or pathogen reservoirs [44]) or institutions (such as schools [45], hospitals [46] or markets [47]). This diverse list shows two things: the complex microcosm of urbanism and urbanisation (including an explicit inclusion of wider urban footprints that embrace suburbia and peri-urban agglomerations), and the enormously wide scope of practice and scholarship of the health/ecosystem connection in this urban universe. The team that has committed itself to further development of One Urban Health includes most of the different disciplines in this universe, from veterinary epidemiology to anthropology, spirituality studies and cosmology, transport engineering, food systems, political science, architecture and design, and (human) public health – it also depends on partnerships with human and ecological communities and their representatives [48]. Assembling dynamic teams like this, within and beyond the Academy, is in itself no guarantee for successful inter- and transdisciplinarity; indeed, that requires skills, visions and beliefs that are more associated with respect, humility, reciprocity, and -Indigenous- cosmology as outlined by Lambert-Pennington and Saija [49]. In order to reconcile the complexity of the urban ecosystem and human health and wellbeing interface, an important transformative gaze would embrace a uniquely human construct in that admittedly deeply anthropocene environment: governance.

**Fig. 1:**
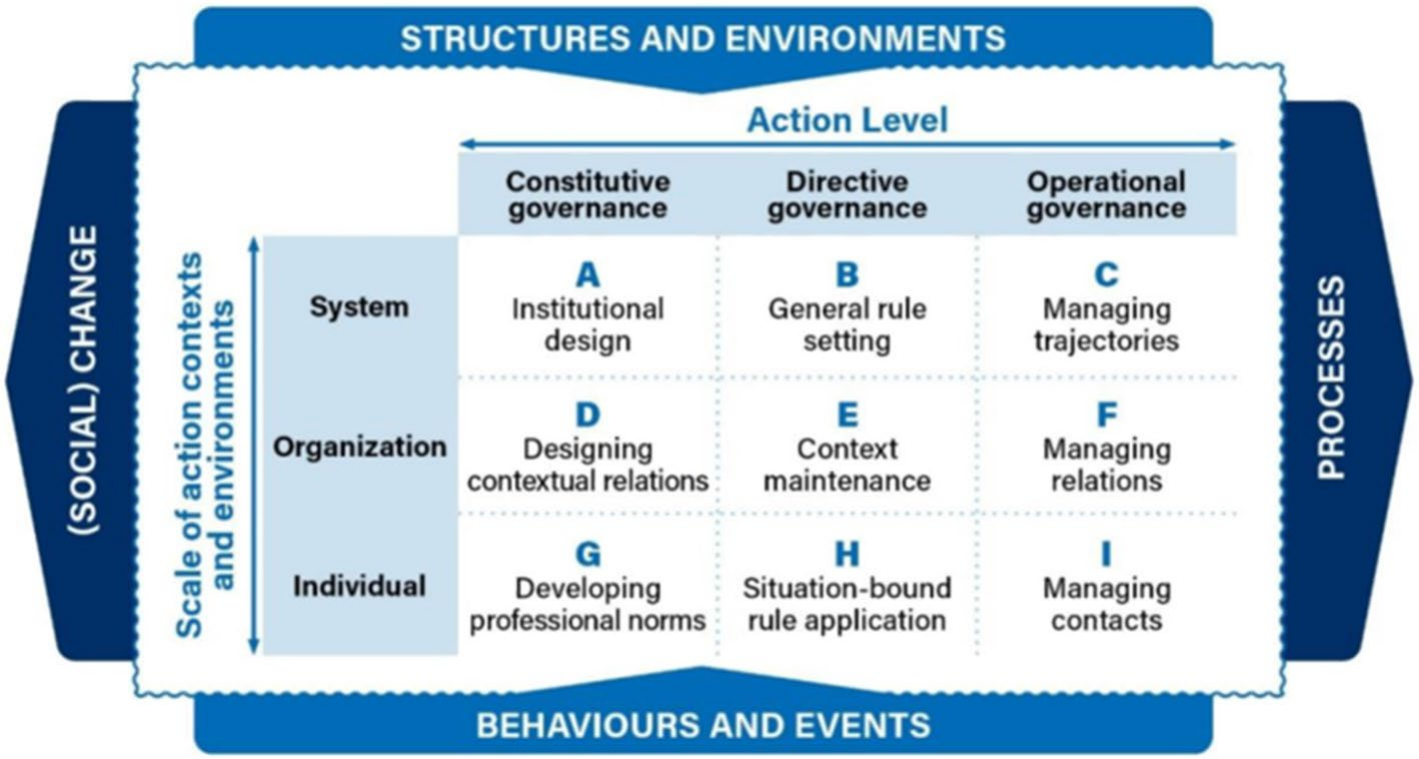
Multi-level governance at the interface of action levels and scales of contexts and environments based on Hill and Hupe [35, 52]

The study of governance is, by some, deemed as notoriously messy. Different schools of thought, lenses and even paradigms abound. A popular (mis)conception is that governance is all about the art and science of *state* governing. Fukuyama, for instance, defines governance as ‘*a government's ability to make and enforce rules, and to deliver services, regardless of whether that government is democratic or not*.’ [50] In the body of literature about health governance (or governance for health), on the other hand, the field is considered multisectoral, multilevel, messy, and embracing community and corporate interests and actions as well as a public policy dimension [51]. This view honours research traditions in network governance and recognises the complex relationships between civil society, the corporate world and the institutional/public sector. Hill and Hupe [35, 52] more proactively embrace these multi-level and reciprocal, institutional and personal relationships and argue that effective (public) policy development and implementation depend in fact on appropriately appreciated and well-managed multi-level governance parameters [52] (Fig. 1). We acknowledge that their 3 × 3 matrix (with cells A through I) is embedded in spatially, cognitively and temporally dynamic contexts and play out in ranges between structures and environments vs behaviours and events on the one hand, and social change processes from global disruption to community action on the other (see also, for scenarios of such perturbations, *Horizons de politique Canada* as per reference [5]). A One Urban Health multi-level governance approach is therefore necessarily embedded in worldviews, community and urban diversity.

Their view of the complex nature of governance can also be simply framed as ‘how we do things around here’ [53]. That perspective honours the implicit as well as explicit nature of rules and institutions involved in the parameters and boundaries of the possible and feasible in shaping collective choice and action – which is, in fact, an appropriate definition of policy-making: ‘*all the processes of conflict, cooperation and negotiation in taking decisions about how resources are to be owned, used, produced and distributed. Inevitably, the contours of politics are framed by the inherited institutional environment (both formal and informal), by the political culture and by the differing degrees and forms of power, which participants bring to the process, and by their interests and ideologies*.’ [36].

Understanding the interface between ecocentric One Health and anthropocentric urbanism through a governance perspective requires a further deliberation and operationalisation of settings. In order to make our One Urban Health approach as tangible as possible, we take settings to be localised places [54] – with a particular territorial relationality and meaning. Places are not just spaces – places are imbued with meaning that is given to it by the human experience and our engagements with the social, natural and physical world. A place-based approach to understanding (governance) processes for ecological and human health in the urban environment is critical: it is precisely *place* that localises actors and actants (and not for nothing we choose Latour’s [55, 56] actor-network language here…). One key – sometimes unfortunate – characteristic of cities is that they all have buildings and streets. Doxiadis’ Science of Settlement [57] provides an analytical and visionary perspective on what he calls ‘shells’ (buildings) and ‘networks’ (including streets). They are the most prominent expression of place: *public* space. In observing this prominence, we build on the seminal and unique pronouncements of urban scholars such as Jacobs [58], and transdisciplinary pracademics such as Duhl [59].

So – if we wish to study the governance of human and ecosystem health in cities, we find that we must ground our transformative research and practice in those very streets. They are the virtual as well as hardware connectors for the urban fabric.

## Participation in street-based One Urban Health

In political science as well as urban planning, the street has already acquired both scholarly and activist prominence. This ranges from a recognition that participation can come in many guises (e.g., Arnstein’s Ladder [60] and Davidson’s Wheel [61]), to an observation that real (policy) change starts in the street: Lipsky [62] was the first to recognise that in spite of all the academic hoopla around political science and policy analyses, ‘*policy implementation in the end comes down to the people [(the street-level bureaucrats)] who actually implement it*.’ And in the process, these street-level bureaucrats bend, amend and morph ‘official’ policy guidance. They embrace disruption. Lipsky and his adherents have built a vast and worthwhile legacy that includes perspectives on policy learning and implementation, and not just at the street level [63]. The importance of the street as a focus of practice and research is further elevated by Corburn: he shows that urban design actually needs to involve, and is driven by, community interests [64]. Where these are ignored, cities start to become worse, rather than better places. In light of the above definition of One Urban Health, we even argue that community interests expressly include recognition of and advocacy for nature’s parameters, including their physical representations (slopes and elevations, rivers, urban meadows and bushland) and its connected life. The connective tissue (street) seems relatively simple: to pursue an accessible open urban commons that generates and sustains, through inspired place-making, social capital [65].

This, of course, includes (or rather, is preceded and is surrounded by) sustainable urban ecosystems with what is called blue (water) and green (plant) health dimensions [66]. But as virtually every city-dweller would observe, the opposition to such salutogenic and sensible cities seems immense and perhaps occasionally even obscene. Most cities are slaves to automobility – with roads and streets quite contrary to health making, inclusive and balanced ecosystems. It is helpful, therefore, to distinguish between roads and streets. *Roads* are connecting pieces of infrastructure for (auto)mobility, whereas *streets* have a communal-public function – they are critical parts of public life such as housing, playing, office working, etc.

A set of transdisciplinary tools and institutions that squarely sit at that street level has emerged. They are street-level laboratories in real-world environments. In particular the rhetoric of the ‘Living Lab’ has become popular. McCrory and colleagues [67] have generated a first inventory, from a sustainability science perspective, of such efforts (see Table 1):

**Table 1:** Conceptual delineation of sustainability-oriented labs as per McCrory et al. [43]

Lab concept	Description	Central Analytical constructs
Urban Living Lab	Governance instrument with a focus on the urban; prioritises geographical embeddedness, experimentation and learning, participation and user involvement, leadership and ownership, and evaluation and refinement	Co-creation Governance Experimentation
Living Lab	A pragmatic, user-centred innovation approach and environment; innovation and design process; co-creation of tech; products, services and ways of living, technology lifecycle	User needs Co-creation Usability Value User innovation
Real-World Lab	Transformative transdisciplinary research approach; real-world problems and contexts; intervention object and study subject; co-design, co-production and co-evaluation	Transdisciplinarity Co-production Learning Experimentation
Evolutionary Learning Lab	A systems-based approach to understand and respond to complex issues; process as well as a setting; test mental models	Systems thinking Mental models Complexity Leverage points
Urban Transition Lab/Transition Management	Transition governance by experimentation. Deliberate process towards the governance of systemic change, including visioning, agenda setting, experimentation and learning	Experimentation Complexity Selective participation Governance Power
Change Laboratory	Seeking transformation of cultural activity systems. A place and a process, including problem analysis and solution development in contexts	Expansive learning Double Stimuli Contradictions
Transformation Lab (T-Lab)	Interactive, participatory innovation spaces that allow for experimentation with new social-ecological technological system configurations and sustainability pathways	Resilience Adaptation Agency
Other	Experimental spaces, urban planning processes, learning environments	Non-overlapping constructs

It appears that, should we pursue an activist inclusive urban transformation agenda with an ecocentric (rather than anthropocentric) perspective, that a Real-World Lab or Urban Transition Lab would be a most prominent model to pursue. Rather than merely basing a One Urban Health R&D enterprise in the street and wait for the world to change around it (which would be the approach of the Living Lab, or Natural Experiment), Real-World Labs and Transition Labs actively seek the framing and implementation of interventions for change. Our current work includes, for instance, Play Streets and School Streets [68], the design of safe pedestrian environments [69], the introduction of grazing animals in urban parks [70], and activist engagement in creating ‘sponge cities’ that absorb and realign natural and social risk [71].

**Fig. 2:**
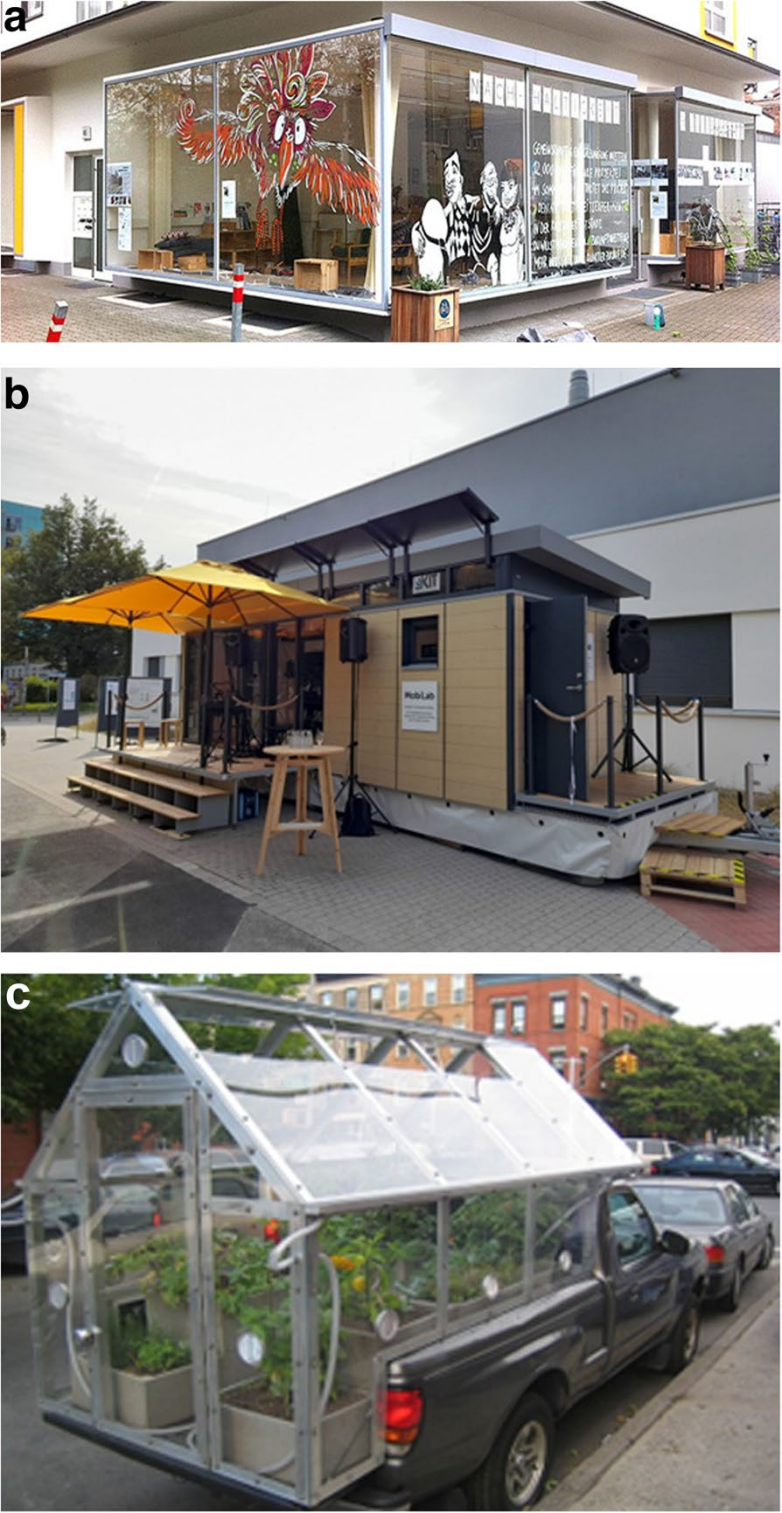
The shopfront (**A**) and mobile (**B**) Real World Labs in Karlsruhe. An example of a mobile Real World Lab as community garden on the USA East Coast (**C**)

The European experience provides guidance towards what such interventionist change laboratories would *physically* look like. At the Karlsruhe Institute of Technology (KIT) in Germany, Real-World Labs (‘Reallabore’) are integral to the academic (research, teaching and service) agendas. Over a period of years, the leadership of KIT has enabled and facilitated the establishment of shop-front entities that are looking and reaching out in co-production with the surrounding community. Where no permanent shopfront could be found or made available, KIT has developed mobile ‘laboratories’ (Fig. 2) [72].

These physical entities serve a clear socio-ecological community development vision that aligns with the demand to work at the interface between communities – meeting in the street. They are catalysts – or nuclei – in the initiation and growth of social and physical environments. In a recent UN—Habitat guidance paper [73], the evidence for the health-making nature of public spaces and places (including streets) in urban environments is convincingly compiled. But what the *text* does not (and the graphic *illustrations* of the document do show profoundly), demonstrate is that in order for public space to be fertile ground for spatial and social development, it needs to have an initial physical benchmark or pivot. The various photographs in the guidance document show such things as shade (a market parasol) and protection (by numbers), boats and floats, seating, colour arrangements, and creative interfaces between nature and culture such as gardens or cycleways. Such pivots create ‘*nucleation*’: starting points for community development and change; a phenomenon documented universally since the start of human and urban evolution, in archaeology [74], and in social movement research [75].

We propose for the Canadian context the deliberate establishment of nucleation catalysts: (community and ecological) health R&D hubs – in Québecois, *Les Dépanneurs de Recherche et Bien-être (Research and wellbeing corner shops)* that can be deployed with versality, including in mobile form.

## The corner shop for One Urban Health

The Canadian province of Québec has over time developed a unique version of a nucleus, known elsewhere as ‘the corner shop’ (Britain) or ‘Milk Bar’ (Australia). In some jurisdictions these places evolved around wells or pumps (e.g., in Africa) or standing-only breakfast facilities for a quick espresso (Italy) or croissant (France). Such community hubs are occasionally gendered (e.g., barbers for men, hair salons for women) or activist (e.g., the ‘Science Shop’ [76] to bring knowledge to the people). Our R&D effort is based in Montréal, the largest city of the Canadian province of Québec. The above-mentioned ‘nucleation’ must be culturally embedded and appropriate. In Québec, the emergency shopping facility on virtually every urban street corner is called a ‘*dépanneur*’. The word comes from the noun ‘*panne*’ – a process breakdown, or bout of bad luck. To address such an unfortunate event, one can ‘*dé-panner*’ (verb – *to fix*). Such quick-fix places have thus become ‘*dépanneurs*’ [77]. In the eyes of many, these quick-fixes mainly peddle cheap alcohol, lottery tickets, tobacco and seedy magazines and have little or no health dimension. But as suppliers of butter, milk, staples and pieces of twine, eggs and other basic needs (such as shelter and warmth during a sudden blizzard) they certainly contribute to community wellbeing.

The *Dépanneurs de recherche et bien-être* would serve to democratise (scientific) research by bringing it down from its pedestal and giving it back to citizens and their ecosystems. Applied to One Urban Health issues, these places would enable citizens and researchers to reconnect with each other, but also with their territory and their physical environment. In some conceptualisations we have scoped mobile community gardens (Fig. 2C) which would reconnect with nature (however artificial) even in harsh winter climates like Montreal’s. In concrete terms, the existence of these – mobile and street-based/facing—sites would enable citizens to become research owners, even leaders, by working through research questions linked to their living environment—their urban ecosystem -, prioritising these questions, participating in the development of methodologies and playing a leading role in the interpretation of results and the mobilisation of knowledge.

Universities across Montreal – and in particular the Université de Montréal – have a strong track record of working with (or being driven by) communities. This happens both in largely informal and unchecked environments (e.g., greening lane ways [78] as guerilla gardening or ecological activism [79]) but also in the more formal consultation processes organised by lower-level governments and non-governmental organizations (NGOs) [80]. Further, building on the rich community-based and creative street artistry history of the Quebecois (home to Cirque du Soleil and an annual mural festival) and following the imperative to use the street as research infrastructure we propose to deploy mobile engagement facilities for intervention research across Montreal (Fig. 2C).

These mobile establishments are evidence-based in their conceptualisation and purpose, but would become not just centres for street-driven ecological and community R&D. They deserve to be assessed, tested and hypothesised themselves within a growing global network of transdisciplinary place-based change efforts [81].

## Evolution and implementation

A summary so far: we seek to integrate a more ecocentric and less anthropocentric view of One Health in urban environments. Such a gaze will be explicitly and deliberately transdisciplinary. Our key parameter of transdisciplinarity is that real world, ecocentric issues drive our agendas, concepts and practices. Ecocentric city realities exist predominantly in the physical connectors of the urban fabric which give meaning to life: streets and green fringes as well as gardens [82]. It is in those streets where our transdisciplinarity needs to take shape and is transformed. Urban archeology (and meteorology) demonstrates that small grains of physical difference (nuclei) can start such transformations. In Québec these important nuclei already exist (‘*Dépanneurs*’) but not for transdisciplinary ecocentric urban realignments which we have called ‘One Urban Health’. We have made an argument why mobile versions of *Dépanneurs de Recherche et Bien-être* can be postulated to have greater and more effective human and ecological community reach.

In the remainder we will reflect on practical steps that can be taken in the Québecois context, that would be pertinent to One Urban Health street-based hubs worldwide as well.

Innovation is often driven by serendipity. Not only is Québec home to Dépanneurs, it is also very fertile ground for any kind of novel interface between science, society and nature [83, 84]. The CERC has become integral to a number of citizen science and boundary-spanning institutional civil society efforts, and has had the ability to (co)lead innovations in that area, including with practical deployment of the Scottish Place Tool [85] for field testing of the proposal. This preliminarily validated the sensibility and feasibility of the approach. A next step is to work with community and activist partners (like the community consultation network *table des concertations montréalaises* [86]) and design colleagues to run a series of charettes (in modern days sometimes called a ‘hackathon’ [87]). Charettes are powerful tools for collaborative design approaches and therefore foundations for transdisciplinarity – but only when participants are purposely engaged from a wide range of communities, sectors and disciplines, and longer range perspectives are (pro)actively developed and managed with this group (see, e.g., Lambert-Pennington & Saija [49]). They will be aiming to add further validation, shape people and ecological support, define the physical and moral dimensions of the *Dépanneurs*, and to assess the long-term ‘fundability’ and resilience of its functional presence in Québec.

## Data Availability

No datasets were generated or analysed during the current study.

## Abbreviations

ANT: Actor-Network Theory
CERC OUH: Canada Excellence Research Chair *One Urban Health*
FAO: Food and Agriculture Organisation of the United Nations
KIT: Karlsruhe Institute of Technology
NGO: Non-governmental organisations
NPI: Non-pharmaceutical interventions
OHHLEP: One Health High-Level Expert Panel
R&D: Research and development
UN: United Nations
UNEP: United Nations Environment Programme
WHO: World Health Organisation
WOAH: World Organisation for Animal Health
